# Acute effects of delta-9-tetrahydrocannabinol, cannabidiol and their combination on facial emotion recognition: A randomised, double-blind, placebo-controlled study in cannabis users

**DOI:** 10.1016/j.euroneuro.2014.11.014

**Published:** 2015-03

**Authors:** Chandni Hindocha, Tom P. Freeman, Grainne Schafer, Chelsea Gardener, Ravi K. Das, Celia J.A. Morgan, H. Valerie Curran

**Affiliations:** a Clinical Psychopharmacology Unit, University College London, United Kingdom; b Department of Psychology, University of Exeter, United Kingdom

**Keywords:** Endocannabinoid system, Emotional processing, Schizotypy, Δ9-tetrahydrocannabinol (THC), Cannabidiol (CBD)

## Abstract

Acute administration of the primary psychoactive constituent of cannabis, Δ-9-tetrahydrocannabinol (THC), impairs human facial affect recognition, implicating the endocannabinoid system in emotional processing. Another main constituent of cannabis, cannabidiol (CBD), has seemingly opposite functional effects on the brain. This study aimed to determine the effects of THC and CBD, both alone and in combination on emotional facial affect recognition. 48 volunteers, selected for high and low frequency of cannabis use and schizotypy, were administered, THC (8 mg), CBD (16 mg), THC+CBD (8 mg+16 mg) and placebo, by inhalation, in a 4-way, double-blind, placebo-controlled crossover design. They completed an emotional facial affect recognition task including fearful, angry, happy, sad, surprise and disgust faces varying in intensity from 20% to 100%. A visual analogue scale (VAS) of feeling ‘stoned’ was also completed. In comparison to placebo, CBD improved emotional facial affect recognition at 60% emotional intensity; THC was detrimental to the recognition of ambiguous faces of 40% intensity. The combination of THC+CBD produced no impairment. Relative to placebo, both THC alone and combined THC+CBD equally increased feelings of being ‘stoned’. CBD did not influence feelings of ‘stoned’. No effects of frequency of use or schizotypy were found. In conclusion, CBD improves recognition of emotional facial affect and attenuates the impairment induced by THC. This is the first human study examining the effects of different cannabinoids on emotional processing. It provides preliminary evidence that different pharmacological agents acting upon the endocannabinoid system can both improve and impair recognition of emotional faces.

## 1 Introduction

Decoding basic human emotional expressions is a key adaptive skill that allows for the prediction of future behaviour and is essential for social communication, interpersonal relationships and mental health (Carton et al., 1999). Impairments in the perception of emotional expression have been reported in depression, anxiety, schizophrenia (Phillips et al., 2003) and in users of a number of drugs (Goldstein and Volkow, 2011) including cannabis (Platt et al., 2010; Hindocha et al., 2014).

Acutely, cannabis and its main active ingredient, Δ-9-tetrahydrocannabinol (THC) produce euphoria and cognitive impairments (Curran et al., 2002; Fernández-Serrano et al., 2011) alongside transient negative emotional states such as anxiety and paranoia (D׳Souza et al., 2004). These emotion-based changes suggest the endocannabinoid system is involved with emotional processing. Cannabinoid receptors are important in processing emotional material (Marsicano et al., 2002) and are abundant in limbic regions including the amygdala, cingulate cortex and hippocampus, as well as the frontal cortex (Pamplona and Takahashi, 2012). Cannabis consists of over 100 cannabinoids, the two most abundant being THC, a partial agonist at the CB1 receptor (Pertwee, 2008) and cannabidiol (CBD) which has a complex mode of action involving several receptor, re-uptake and enzymatic proteins. It also inhibits the reuptake and hydrolysis of the endocannabinoid, anandamide (Pertwee, 2008). These cannabinoids have opposing psychological and emotional properties, for example, whilst THC can induce acute anxiety (Martin-Santos et al., 2012) and amnesic effects (Curran et al., 2002), CBD may have some anxiolytic effects (Guimaraes et al., 1990) and has been shown to block THC-induced memory impairments (Morgan et al., 2010b; Englund et al., 2013).

In experienced cannabis users, THC impairs the recognition of unambiguous (100% emotional intensity) faces displaying fear and anger in a dose-dependent manner (Ballard et al., 2012) and impairs accuracy scores for negative emotional faces only (Bossong et al., 2013). Three functional neuroimaging studies have investigated the involvement of the endocannabinoid system in human emotional processing with mixed results. Acute THC administration (7.5 mg oral) has been shown to decrease amygdala BOLD (blood-oxygen level dependent) response to threatening faces (Phan et al., 2008). Using the same emotional face discrimination task, Bossong et al. (2013) found 8 mg pulmonary THC decreased activity in a network including the amygdala, hippocampus, parietal and prefrontal gyrus in response to threatening faces; however activity in this network increased during non-threatening faces. Fusar-Poli et al. (2009) conducted a gender discrimination task with negative faces and found THC (10 mg oral) increased activity in the precuneus and primary motor cortex and decreased activity in the right inferior frontal gyrus, superior temporal gyrus and left medial frontal gyrus.

CBD has rarely been studied in the context of emotional processing and has not yet been shown to have behavioural effects. Nevertheless, CBD has been hypothesised to reduce anxiety through an anterior cingulate cortex (ACC) mediated attenuation of the amygdala, in response to fearful and angry faces (Fusar-Poli et al., 2009; Kowal et al., 2013). In support of this, CBD disrupts the effective connectivity between ACC and amygdala (Fusar-Poli et al., 2010) and produces opposite effects to THC, on amygdala BOLD activation and skin conductance response (SCR) fluctuations when viewing fearful faces (Bhattacharyya et al., 2010). The opposing effects of THC and CBD on BOLD signal and SCRs during this emotional processing task suggest whilst THC may impair emotional recognition, CBD may tentatively be expected to improve it, relative to placebo.

Very little is known about how THC and CBD interact. Although THC and CBD are the most abundant cannabinoids found in the cannabis plant, high THC varieties are most common. Nevertheless, the ratio of CBD:THC varies hugely (e.g. 0.0–4.3; Freeman et al., 2014). In smoked cannabis, THC and CBD are typically both present, high levels of CBD appear to protect against some psychotomimetic, anxiogenic, cognitive impairing, and dependence forming effects of THC (Morgan and Curran, 2008; Morgan et al., 2010a, 2010b, 2012). We therefore hypothesised acute THC would impair facial affect recognition, CBD alone might enhance it and in combination, CBD might protect against THC-induced impairments.

The acute effects of cannabis also depend on individual vulnerabilities to its harmful effects. Habitual cannabis use is associated with impairments in emotional processing; heavy users show impaired accuracy when non-intoxicated (Platt et al., 2010; Hindocha et al., 2014). Simultaneously, heavy cannabis users show tolerance to cognitive impairment following acute THC administration (D׳Souza et al., 2008). Evidence for the association between adolescents using cannabis and psychosis continues to accumulate. Longitudinal population-based studies show a two-fold increase in risk of psychotic illness (Moore et al., 2007; Di Forti et al., 2007). Schizotypy or ‘psychosis-proneness’ has further been associated with deficits in emotional processing (Germaine and Hooker, 2011; Edwards et al., 2001) and enhanced acute response to cannabis (Mason et al., 2009). This study aimed to determine the effects of THC and CBD, both alone and in combination on emotional facial affect recognition. However, given the significant inter-relationship between emotional recognition, psychosis-proneness and cannabis use, we aimed to explore how variations in frequency of use and schizotypy might modulate an individual׳s acute response to cannabinoids. Therefore we recruited 48 cannabis users, half of whom used the drug heavily and half used it recreationally. In each of these two groups, half the participants scored high in schizotypy and half low in schizotypy. All 48 participants were tested on 4 separate days; each being administered a single dose of inhaled THC, CBD, combined THC+CBD and placebo.

## 2 Experimental procedures

### 2.1 Participants

Participants were recruited as 24 light (1–24 days per month) and 24 heavy (25+ days per month) cannabis users following the criteria of Morgan et al. (2012). 50% of each of these groups scored high, and 50% scored low, in schizotypy (Schizotypal Proneness Questionnaire score) and were selected from the bottom and top quartiles of our previous study large-scale study of over 400 cannabis users (Morgan et al., 2012). They were recruited as such to systematically investigate the effects of schizotypy and tolerance on the interaction between the endogenous cannabinoid system and emotional processing. However, in the 6 months to 2 years between testing on the two experiments, some participants changed their level of cannabis use and schizotypy scores (reported in Table 1). Participants were matched for age and premorbid verbal intelligence (as measures by the spot the word task) across heavy and light users. Inclusion criteria were: (i) self-reported abstinence from cannabis, other drug and alcohol use for 24 h prior to each test day; (ii) fluent in English, (iii) normal or corrected to normal vision. Exclusion criteria were: current self-reported (i) respiratory health problems or physical health problems, (ii) pregnancy or the risk of being pregnant, (iii) clinically diagnosed learning impairments, (iv) clinically diagnosed schizophrenia/psychosis or substance abuse problems, and (v) illicit drug use other than cannabis more than once a week.

### 2.2 Design

A randomised, double-blind, placebo controlled study was used to compare the acute effects of inhaled THC (8 mg), CBD (16 mg) and their combination (8 mg THC+16 mg CBD) with placebo (ethanol vehicle). Cannabinoids were formulated in alcohol solution and were purchased from STI Pharmaceuticals (Brentwood, Essex, UK). Four groups of individuals took part in this balanced crossover design: group (low schizotypy, light cannabis users (LS-L); low schizotypy, heavy users (LS-H); high schizotypy, light users (HS-L); high schizotypy, heavy users (HS-H)). *N*=12 per experimental group was chosen to detect THC-induced (compared to placebo) impairment in memory at a power of 0.83 (Curran et al., 2002). Order of drug administration was randomised using a partial Latin square, resulting in 12 different combinations. Participants had all previously partaken in a naturalistic study of cannabis in 400 young people and had consented to be contacted about subsequent studies. Participants were screened for eligibility via a brief telephone call. Testing sessions occurred on four occasions each separated by a one week wash-out to minimise carry-over effects (>3 times elimination half-life of THC; D׳Souza et al., 2008). Participants completed baseline questionnaires before, and then commencing 10 min after drug administration. Four versions of the emotional processing task were administered. Version was counterbalanced. The test battery took approximately 1.5 h on each test day and included other tasks not reported. Participants were reimbursed £120 for their time on the last day and debriefed fully. All participants provided written, informed consent on each occasion. Ethical approval was given by the UCL Graduate School Ethics Committee.

### 2.3 Assessments

#### 2.3.1 Emotional processing task

This computer based task assessed emotional facial affect recognition and is described in more detail in Hindocha et al. (2014). Faces were taken from the NimStim Face Stimulus Set (Tottenham et al., 2009) and were created with Fotomorph 5.2. Two male and two female faces were used to portray the 6 basic emotions – happiness, sadness, anger, disgust, fearful, surprise and neutrality. Each face varied in the degree of intensity it portrayed from 0% (neutral) to 100% on a continuum in 10% increments. Approximately 200 fucidial markers were positioned on the actors׳ neutral face around main facial features. The markers were then placed onto the actor׳s emotional expression and morphed between the full emotion (100%) and neutral (0%) in 10% increments and stills were taken at these points (Harmer et al., 2003). These were combined into 20% increments to give 5 levels of intensity of 20%, 40%, 60%, 80% and 100% for each emotion. In total, 250 stimuli were shown, 40 faces/emotion and 10 neutral faces (100% intensity). The primary outcome variable was recognition accuracy calculated as percentage of correctly identified emotions.

**Figure 1:**
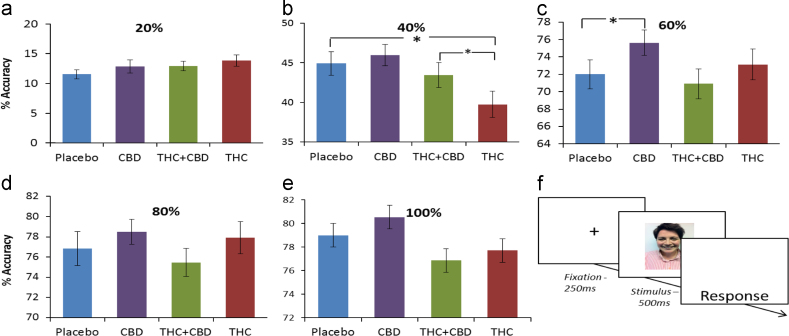
Percentage accuracy for each intensity, over all emotions and single trial design. Asterisks indicate a significant drug×intensity interaction. (a) Percentage accuracy at 20%, (b) significant contrast between THC and Placebo at 40%, (c) significant contrast between CBD and Placebo at 60%, (d) percentage accuracy at 80%, (e) percentage accuracy at 100%, and (f) design of a single trial in the emotional processing task. Error bars represent standard error of the mean (±SEM).

All stimuli were presented in the centre of a white background. Participants indicated the correct facial expression as quickly and as accurately as possible with no feedback except on the 7 practice trials at the beginning of the task. Following the offset of a black fixation cross (250 ms) participants were presented with a single face for 500 ms and then pressed a labelled key (response) corresponding to an emotion (see Figure 1f). Their response led to the next stimulus onset. All responses were self-paced. Trials were randomised apart from the restriction that two faces of one emotion were not shown more than twice in succession.

#### 2.3.2 Questionnaire measures

Before drug administration participants completed the Beck Depression Inventory (BDI; Beck et al., 1961), the trait scale of the Spielberger State Trait Anxiety Inventory (STAI; Spielberger et al., 1983), the Schizotypal Proneness Questionnaire (SPQ; Raine, 1991) and the Spot the Word Test – which correlates highly with premorbid verbal intelligence (STWT; Baddeley et al., 1993). Additionally, participants completed 10-point VAS scales of subjective effects of ‘stoned’, anchored at ‘not stoned’ and ‘extremely stoned’, VAS ‘anxiety’ anchored at ‘no anxiety’ and ‘very severe anxiety’, VAS ‘alert’ anchored at ‘not alert’ and ‘extremely alert’, VAS ‘happy’, anchored at ‘sad’ and ‘happy’ at pre-drug administration (−15 min), 2 min after drug administration (+2 min), and then every 30 min (+30 min, +60 min, +90 min and +120 min).

#### 2.3.3 Drug administration

Cannabinoids and placebo (ethanol vehicle) were administered using a Volcano Medic Vaporisor (Storz and Bickel, Tuttlingen, Germany); a safe, effective and replicable method of intrapulmonary cannabinoid administration (Hazekamp et al., 2006; Abrams et al., 2007). This administration method was preferred as it overcomes the variable bioavailability of oral administration and bypasses inhalation of toxic compounds as per burning cannabis (Van Hell et al., 2011). Moreover, final pulmonary, plasma and subjective levels are equivalent to smoked cannabis (Hazekamp et al., 2006; Abrams et al., 2007). Cannabinoid doses were selected on the basis of prior research in which 8 mg THC was vaporised into a single balloon using a Volcano device (Bossong et al., 2009). In a sample of incidental cannabis users, this dose elicited robust psychotomimetic and subjective effects, and produced a reduction of [^11^C] raclopride binding in the ventral stritum, consistent with an increase of dopamine (Bossong et al., 2009). This dose was further based on the pharmacokinetic/pharmacodynamic profile of inhaled THC (Zuurman et al., 2008). We have previously reported psychophysiological and behavioural effects following 32 mg CBD (Das et al., 2013). In this study a dose of 16 mg was chosen to create a CBD:THC ratio of 2:1, reflecting the upper limit (mean +3 SD) found in high CBD/low THC cannabis preparations (Freeman et al.,2014). 54% (±8%) of the loaded dose is delivered in the administration balloon (Hazekamp et al., 2006), thus the aim was to deliver the maximum total amount of cannabinoids vaporised such that 5 mg THC and 10 mg CBD would be inhaled. Participants were given a practise balloon to familiarise themselves with the procedure before any drug administration occurred. On drug days, 8 mg THC and/or 16 mg CBD dissolved in ethanol were administered on a 10-s inhalation cycle wherein the participant was instructed to first fully exhale, next fully inhale from the balloon, hold their breath for 10 s and then fully exhale; this was repeated until the balloon was empty (Bossong et al., 2009). A single balloon was filled (as per guidelines from Hazekamp (2009)), covered with an opaque bag, and administered by an independent researcher to maintain blinding of the experimenter collecting behavioural data and participant.

### 2.4 Statistical analyses

Between group analyses were conducted in the groups that participants were recruited as. Demographics and scores on questionnaires were analysed using ANOVAs with two between-subjects variables (frequency of use, schizotypy). When variables showed a main effect or interaction this was explored with post-hoc tests (Bonferroni-corrected). For the emotional processing task, a mixed model repeated measures ANOVA was carried out on mean percentage accuracy. This included within subjects-factors of drug (placebo, THC, THC+CBD, CBD), emotion (angry, disgust, fear, happy, sad, surprise) and intensity (20%, 40%, 60%, 80%, 100%) and two between subjects factors (frequency of use – light/heavy; schizotypy – high/low). Simple effects analyses of each drug condition in comparison to placebo were used to explore drug main effects and interactions (Bonferroni corrected). Other interactions were explored with separate ANOVAs and Bonferroni-corrected *t*-tests. One Participant’s data (HS-H group) was removed from the emotional processing task only for being >3 standard deviations away from the mean in >50% of trials. This participant remains in all analyses that do not involve emotional processing data. Further exploration included between-group differences in gender and of carry-over effects. Gender was included as a between-subjects factor in the ANOVA as it has previously been shown that males show a lower accuracy on this task (Hindocha et al., 2014). Carry-over effects were explored with mixed model ANOVAs conducted separately for each drug, including the within subjects factor of intensity (20%, 40%, 60%, 80%, 100%) an additional between subjects factor of ‘occasion’ (1, 2, 3, 4). Schizotypy and frequency of use were not entered into these models in order to minimise the number of factors in this exploratory analysis.

Cannabinoid effects on VAS ‘stoned’, VAS ‘anxiety’, VAS ‘alert’, and VAS ‘happy’ were analysed using a repeated measures ANOVA with Drug (Placebo, THC, THC+CBD, CBD) and Time (−15, +2, +30, +60, +90, +120) as within-subjects׳ factors and between-subject׳ factors of frequency of use and schizotypy. Interactions were followed up with separate Bonferroni corrected ANOVAs and pairwise comparisons as necessary (*p*=0.008). Greenhouse Geisser corrections (degrees of freedom rounded to the nearest integer) were used when assumptions of sphericity were violated for all analyses.

## 3 Results

### 3.1 Demographics (Table 1)

Groups did not differ in age (*F*_(3,44)_=2.54, *p*=0.070), gender, scores on the spot the word task (*F*_(3,44)_=0.80, *p*=0.490), last use of cannabis, or number of year’s cannabis had been used (*F*_(3,44)_=0.67, *p*=0.580). There was a main effect of schizotypy on scores on the SPQ (*F*_(1,44)_=12.47, *p*<0.001), BDI (*F*_(1,43)_=14.70, *p*<0.001) and STAI (*F*_(1,44)_=9.05, *p*=0.004) where the high schizotypy group had higher scores than the low schizotypy group for each measure. Light and heavy users of cannabis differed on the time to smoke 3.5 g of cannabis (*F*_(1,46)_=8.22, *p*=0.006) and on number of days per month they used cannabis (*F*_(1,44)_=8.54, *p*=0.005) where heavy users smoked 3.5 g in less days and used cannabis on more days per month than light users.

### 3.2 Emotional processing task

**Figure 2:**
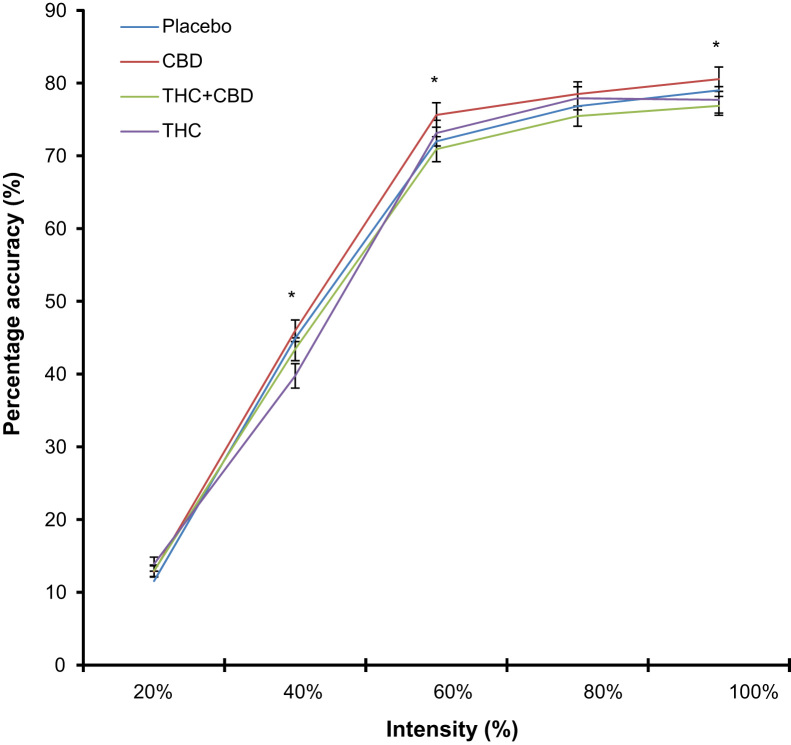
(a) Recognition of all emotions over the different intensities. Asterisks׳ illustrate a significant drug by intensity interaction where at 40%, we found a significant contrast between placebo and THC; at 60%, a significant contrast between placebo and CBD and at 100%, no significant contrast after correction for multiple comparisons.

Repeated measures ANOVA revealed a drug×intensity interaction (*F*_(8,355)_=3.25, *p*=0.001) and an emotion×schizotypy interaction (*F*_(4,170)_=3.02, *p*=0.020). In exploring the drug×intensity interaction we conducted repeated measures ANOVAs collapsing over emotion to compare each drug over different intensities as seen in Figure 1 (20–100%). Figure 2 depicts the increase in performance with increasing intensity. At 40% we found a main effect of drug (*F*_(3,138)_=7.56, *p*<0.001). Simple contrasts showed a significant difference only between THC and placebo (*F*_(1,46)_=16.20, *p*<0.001) with poorer accuracy after THC (Figure 1b); no other contrasts were significant. To test the hypothesis that THC+CBD would protect against the negative effects of THC here, we conducted a paired-samples t-test between THC and THC+CBD at 40% which revealed that participants were more accurate on THC+CBD (*M*=43.52%, SD=10.9%) compared to THC alone (*M*=39.75%, SD=4.51%) (*t*_(46)_=−2.33, *p*=0.024). We also found a main effect of drug at 60% (*F*_(3,138)_=7.56, *p*=0.004). The significant contrast here was CBD vs. placebo (Figure 1c); Participants were significantly more accurate after CBD (*F*_(1,46)_=7.32, *p*=0.010). The effect size for this contrast (partial eta squared=0.137) which is within the moderate range of 0.10–0.30. Finally, at 100%, we found a main effect of drug (*F*_(3,138)_=6.95, *p*<0.001) but no contrasts was significant after correcting for multiple comparisons. No drug differences were found at 20% or 80%.

There were also main effects of drug (*F*_(3,129)_=4.80, *p*=0.003), emotion (*F*_(4,170)_=61.86, *p*<0.001) and intensity (*F*_(2,104)_=2540.89, *p*<0.001). There were no main effects of frequency of use or schizotypy or any other interactions. Simple contrasts revealed that the main effect of drug was reflecting significantly greater accuracy following CBD compared to placebo (*F*_(1,43)_=5.30, *p*=0.026). Equivalent contrasts for THC and THC+CBD vs. placebo were not significant.

The emotion×schizotypy interaction was explored with independent t-tests, collapsing over intensity and drug. After correction for multiple comparisons there were no significant differences between high and low schizotypy groups for any emotion. However, the direction of this interaction suggests the high schizotypy group (*M*=25.93%, SD: 9.89%) were less accurate than the low schizotypy group (*M*=32.15%, SD=9.18%) for fearful faces only.

### 3.3 Exploratory analyses

#### 3.3.1 Gender

There were no main effects or interactions with gender; moreover, this analysis did not alter the main effects or interactions above.

#### 3.3.2 Carry-over effects

Due to the small cell sizes and large number of orders (12 in total), order could not be included as a between-subjects factor. For placebo, THC or CBD, occasion did not interact with accuracy. For the THC+CBD condition, an occasion×accuracy interaction was detected (*F*_(9, 120)_=1.99, *p*=0.044). When Bonferroni corrected, this was no longer significant, however for due diligence, we then coded whether the THC+CBD day was preceded by placebo (or was on the first day), THC or CBD. No interaction was found between performance on the THC+CBD day and the drug occasion it was preceded by.

### 3.4 VAS ‘stoned’ (Figure 3)

Repeated measures ANOVA revealed a drug×time interaction for feelings of ‘stoned’ (*F*_(8,330)_=9.25, *p*<0.001) as well as a main effect of drug (*F*_(3,114)_=15.60, *p*<0.001) and time (*F*_(2,100)_=72.96, *p*<0.001). There was no significant between-subjects׳ main effects or interactions with drug or time. To explore this interaction we conducted ANOVAs for each time-point and found drug main effects at all time-points post drug administration (all *p׳*s≤0.001). At all time points post drug administration, Bonferonni-corrected paired *t*-tests between drug conditions at each time revealed greater ‘stoned’ feelings for THC vs. placebo (all *p׳*s≤0.001) and greater feelings for stoned for THC+CBD vs. Placebo (all *p׳*s≤0.001). Feelings of ‘stoned’ between CBD and placebo did not differ at any time (all *p׳*s>0.05). We found greater feelings of ‘stoned’ for THC compared to CBD at +30, +60, +90 and +120 min (all *p׳*s<0.001) and greater feelings of ‘stoned’ were also found for THC+CBD in comparison to CBD alone at these time points (all *p׳*s<0.001). Finally, THC and THC+CBD produced equivalent ratings of ‘stoned’ across all time points (all *p׳*s>0.05).

### 3.5 VAS ‘anxiety’, VAS ‘alert’ and VAS ‘happy’

Comparable repeated measures ANOVAs were conducted for the remaining VAS scales. VAS ‘anxiety’ revealed a main effect of drug (*F*_(2,95)_=3.05, *p*=0.050), and a main effect of time (*F*_(3,129)_=2.96, *p*=0.030). After correction for multiple comparisons, there was no significant difference between drug conditions. No interaction between drug and time emerged and there was no significant between-subjects׳ main effects or interactions with drug or time. For VAS ‘alert’, the repeated measures ANOVA did not reveal any main effects or interactions apart from a main effect of time (*F*_(5,210)_=22.17, *p*<0.001), for VAS ‘happy’, no main effects or interaction emerged.

### 3.6 Correlations

Correlations were carried out between self-report measures (BDI, STAI, SPQ and STWT) with performance on the emotional processing task across cannabinoid administration, to investigate whether baseline psychological wellbeing was associated with performance accuracy, however no correlations emerged (all *p׳s*>0.05). Moreover, accuracy at 60% between CBD and placebo, and at 40% between THC and placebo did not correlate with SPQ scores or frequency of cannabis use. No correlations emerged between self-reported measures and accuracy on individual emotions collapsed over drug. VAS ‘stoned’, ‘anxiety’, ‘alert’ and ‘happy’ at all time-points did not correlate with accuracy on the emotional processing task or self-reported measures.

## 4 Discussion

This study aimed to investigate the separate and combined effects of THC and CBD on emotional facial affect recognition. This is the first human study to examine the effects of a combination of cannabinoids on emotional processing. We first hypothesised THC would be detrimental to emotional processing (Ballard et al., 2012; Bossong et al., 2013). Second, we hypothesised when CBD was combined with THC, it would protect against the impairments produced by THC (Morgan and Curran, 2008; Morgan et al., 2010a, 2010b, 2012). We found acute administration of cannabinoids altered facial affect recognition and this varied with the intensity of expression the face portrayed, irrespective of emotion. Our most novel finding is in comparison to placebo, acute administration of CBD improves emotional facial affect recognition, at 60% intensity of emotion. THC, in comparison to placebo, significantly impaired the recognition of emotional faces of 40% intensity. Moreover, in tentative support of our hypothesis, the combination of THC with CBD protected against the impairment THC alone produced at 40%. These subtle findings suggest cannabinoids influence emotional processing at levels of intermediate emotional intensity (above and below 50%), and further these effects persist after a conservative correction for multiple comparisons. Above 60%, all participants reach ceiling responses of accuracy.

CBD has known anxiolytic and antipsychotic properties. It has previously been hypothesised CBD exerts its anxiolytic effect through an ACC-mediated attenuation of the amygdala in response to threat-related faces (Fusar-Poli et al., 2009; Kowal et al., 2013). This study provides the first evidence that a single dose of inhaled CBD can produce a behavioural improvement in emotional facial recognition. Furthermore, this was equal across groups of high and low schizotypy and frequency of use. Although only evident at 60% emotional intensity, this suggests, if used clinically, CBD would be equally effective in groups of high and low schizotypy and frequency of cannabis use. This is unlikely to be a product of the change in subjective effects as we found no difference between CBD and placebo on feelings of VAS scales of ‘stoned’, ‘anxiety’, ‘alert’ or ‘happy’. Ratings of ‘stoned’ were not influenced by frequency of use, replicating findings that frequent users cannot be distinguished from healthy controls by euphoric effects (D׳Souza et al., 2008). It should be noted that although the light users reported here were using cannabis at a recreational level (~12 days per month), this was higher than controls in D׳Souza et al. (2008) (~0.16 exposures in the last month) and may still be associated with tolerance. Feelings of ‘stoned’ did not interact with schizotypy, consistent with D׳Souza et al. (2005), who did not find an interaction between euphoric effects of THC and time, in patients with schizophrenia and further consistent with Barkus et al. (2006) who found that the pleasurable effects of cannabis, as measured by the Cannabis Experiences Questionnaire, were not associated with schizotypy.

In experienced cannabis users, Ballard et al. (2012) previously found that 7.5 and 15 mg oral THC impaired recognition of negative faces (100% intensity), in a dose dependent manner in healthy volunteers. Bossong et al. (2013) found that 8 mg inhaled THC reduced accuracy of recognising negative emotional faces. In the present study however, 8 mg inhaled THC consistently decreased accuracy across all emotions but only at 40% emotional intensity. The disparity between these studies may possibly be a result of the sample characteristics, as previous studies have used incidental cannabis users. The reduced ability to interpret ambiguous emotional faces after THC inhalation might increase social risk-taking behaviour and in affective disorders the skewed interpretation of ambiguity can be seen as a causal mechanism in the maintenance of the disorder. In major depression, for example, research clearly shows a reduced recognition to both positive and negative emotional valence which suggests that both recognition accuracy and interpretation of ambiguous faces are important in the aetiology of the disorder (Bourke et al., 2010). Our previous findings with heavy cannabis users, using the same paradigm, show significant non-acute impairments at 40%, 60% and 100% (Hindocha et al., 2014). Thus perhaps, when uncertainty is high, THC seems to impair performance. Cannabis users require more information from a face to discern an emotion (Platt et al., 2010). Therefore, perhaps when the available information is low in cannabis users, THC further impairs one׳s ability to correctly respond to facial affect. The present study was unable to investigate this hypothesis as it was a self-paced task. Future studies should employ a dynamic face processing paradigm to determine if this indeed is the case.

We found that CBD alone can reverse the impairment in faces that are slightly more unambiguous supporting its potential role in the treatment of disorders characterised by impairments in emotional facial affect processing. A recent proposal suggesting that the mood effects of psychotropic drugs are mediated through processing of emotional information in the absence of effects on subjective states (Harmer et al., 2009) might explain why a single dose of CBD improved emotional affect recognition in the absence of any effects on mood (VAS ‘anxiety’). It remains to be established whether drugs such as CBD might be effective in alleviating emotional processing deficits after repeated dosing.

In smoked cannabis, the relative active THC/CBD combination suggests CBD is antagonistic to the psychological and memory impairing effect of THC (Morgan et al., 2010b, 2012; Morgan and Curran, 2008). It is also difficult to identify when the effect of CBD will potentiate or antagonise the effects of THC as there is little evidence about the interaction of these two cannabinoids in humans, especially on cognition and mood. Our exploratory analysis comparing THC alone with THC combined with CBD suggests that when given this combination, participants performed better at 40% then THC alone. Further, we did not find an effect of inhaled THC+CBD compared to placebo. Thus, at this intensity, both conditions performed equally such that CBD seems to normalise emotional processing when combined with THC. Moreover, a combination of THC and CBD produced subjective effects equal to THC alone, replicating our previous finding in smoked cannabis that CBD does not reduce the feeling of ‘stoned’ (Morgan et al., 2010a).

Participants were chosen for high and low frequency of use (Platt et al., 2010; Hindocha et al., 2014) and schizotypy (Morgan et al., 2012); as it was hypothesised that cannabinoids would differentially act on emotional facial affect recognition in groups with varying vulnerabilities to cannabis use. Contrary to this hypothesis, we found no interaction with frequency of use or schizotypy; THC was equally detrimental to emotional recognition in heavy and light cannabis users, with high and low levels of schizotypy. These findings correspond with our previous study (Hindocha et al., 2014) which found that heavy cannabis users show impairment in emotional recognition in comparison to controls, independent of schizotypy. The equivalent performance of light and heavy users suggests the chronic effects of cannabinoids on emotional processing in cannabis users are not dose-dependent but instead may be a feature of cannabis use generally. Future research should employ a prospective design to discern whether these effects are a consequence of cannabis use or pre-dates cannabis use itself. Importantly, CBD was equally effective in this wide variety of cannabis users. If CBD were found to have clinical efficacy for emotion processing deficits, these findings tentatively suggest that its beneficial effects would not be compromised in people with varied heaviness of cannabis use or schizotypy.

The effect sizes of our findings were in the modest range (partial eta squared: 0.1–0.3). Bourke et al., (2012) report, using a similar task, that those with a Major Depressive Episode (42.2% ±0.9) or Social Anxiety Disorder (42.6% ±1.3) are equally accurate in comparison to healthy controls (42.0% ±1.0). Further, they found no interaction between group and emotion for accuracy, and the effects of intensity were similar across the groups. Kohler et al. (2003) found that for mild-intensity faces, healthy controls only perform 4% better than patients with schizophrenia (61.1% vs. 57.1%). In comparison, we report here, at 60% emotional intensity, participants performed 3.6% better on CBD than placebo. Moreover, at 40%, when given THC, participants perform at 5.2% poorer than placebo. This suggests the effects of single dose cannabinoid administration are small, but of similar magnitude to that of clinical populations in previous research (e.g., Kohler et al., 2003). Future research is necessary to determine whether these early experimental results might lead to adequate benefit in patients, either using single or repeated doses of cannabinoids.

This study has several strengths; it used a large sample size in a four-way cross-over and highly controlled laboratory settings. The Volcano Vaporiser method of administering cannabinoids produces similar plasma and pulmonal THC levels in comparison to smoked cannabis cigarettes (Abrams et al., 2007) and delivers between 34% and 69% of the loaded THC (Hazekamp et al., 2006). Our findings of impaired recognition across all emotions are similar to previous data comparing cannabis users to controls (Hindocha et al., 2014, Platt et al., 2010). On the other hand, they differ from previous studies in which drug effects have emerged for specific emotions (e.g. Ballard et al., 2012; Bossong et al., 2013). Although poor recognition of negative and positive emotion has been reported elsewhere (for example Major Depression; Bourke et al., 2010), an alternative explanation for our findings is a non-specific perceptual or attentional effect independent of emotional processing. Future research should therefore employ additional tasks in order to address this issue. An important limitation to note is the lack of THC and CBD plasma measures, which hinders the ability to make conclusions about the THC+CBD group, especially as this is the first human study to assess this combination of cannabinoids on emotional processing. The plasma levels of THC in this study would be equivalent to that of Bossong et al. (2009) which also used 8 mg inhaled THC. Pharmacokinetic/Pharmacodynamic (PK/PD) models (Strougo et al., 2008) were applied to this concentration/time profile of THC in the study by Bossong et al. (2009) and found that 84.5–95.9% of the maximum CNS effects were still present 45–85 min after drug administration. This timescale matches the current study. Further effects of ‘feelings of stoned’ were still reported 120 min after drug administration and were still significant in comparison to placebo. This suggests that CNS effects may be responsible for our current findings. This study found no systematic differences in individuals with high/low frequency of use or schizotypy, there is no reason to assume that these users are not comparable in levels of plasma (or CNS) THC and/or CBD. There were also some changes in ‘trait’ schizotypy and frequency of cannabis use between when participants were recruited and when they were tested for this experiment, therefore we caution readers about making interpretations about the lack of between group effects, which are evident and convincing elsewhere (D׳Souza et al., 2008). When we correlated our key findings with the total schizotypy scores and frequency of use, no correlations emerged which supports the lack of between group effects. Moreover, urine screens were not conducted, so we have limited knowledge about the extent of cannabis and other drug use in between sessions. Future research should attempt to control for this. Participants were texted 24 h before each testing session, reminding them to remain abstinent. They also gave self-reported abstinence of cannabis, alcohol and other drugs for 24 h before testing sessions. If residual effects of drugs were an explanation for this pattern of findings then we would have expected to find systematic ‘frequency of use effects’ across our data, because of recent use in heavy users. Finally, our use of a static version of the face morphing paradigm which may have less ecological face validity than a dynamic face morphing paradigm (Platt et al., 2010).

To our knowledge, this is the first research to show that CBD can improve emotional facial affect processing. Nevertheless, it is important to consider these findings as preliminary. This study used an experimental medicine approach to investigate predictors of vulnerability, with relatively small numbers in each sub-group and a large number of comparisons. Replication of these findings is warranted before their clinical relevance can be fully determined. This study may further be extended by using a range of CBD doses. A potentially fruitful avenue for investigation may be to investigate whether CBD can ameliorate emotional processing deficits that are characteristic of mood disorders such as depression or anxiety, and furthermore, whether CBD might be used as an adjunct to psychological and pharmacological therapies for mood disorders.

### 4.1 Conclusion

In summary, the findings of this study add to previous evidence that acute THC administration reduces cannabis users׳ ability to accurately identify facial emotions. Importantly, this study provides the first behavioural evidence that CBD subtly enhances emotional facial affect recognition and protects against the impairments produced by THC.

## Role of funding source

Funding for this study was provided by the Medical Research Council (MRC); the MRC had no further role in study design; in the collection, analysis and interpretation of data; in the writing of the report; and in the decision to submit the paper for publication.

## Contributors

HVC and CJAM designed the protocol. CJAM, HVC, GS and CG undertook data collection. Cannabinoids were administered by TPF and RKD. CH and TPF undertook statistical analysis and CH wrote the first draft of the manuscript. All authors contributed to and have approved the final manuscript.

## Conflict of interest

No conflict declared.

## Figures and Tables

**Figure 3:**
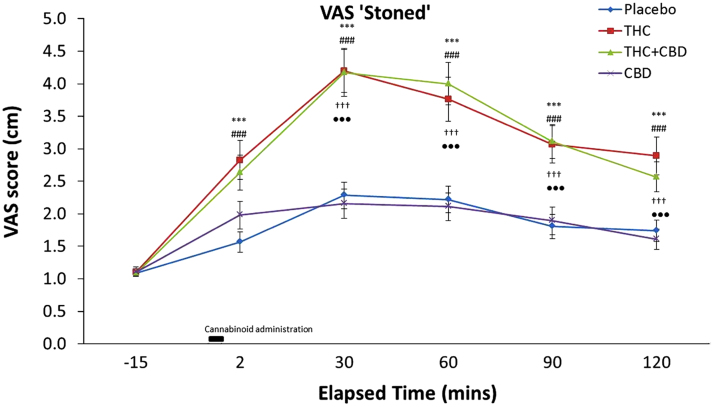
Subjective ratings of feeling ‘stoned’ averaged across all participants for all time points. Significant differences between cannabinoid conditions are indicated as followed: ^⁎⁎⁎^THC vs. placebo (*p*≤0.001); ^♯♯♯^THC+ vs. placebo (*p*≤0.001); ^†††^THC vs. CBD (*P*<0.001); ^•••^THC+CBD vs. CBD (*p*<0.001). Error bars represent standard error mean (±SEM).

**Table 1:** Group demographics (means and SD) and questionnaire data based on recruitment strategy.

	**Light**		**Heavy**	
	**Low schizotypy**	**High schizotypy**	**Low schizotypy**	**High schizotypy**
Age	21.00 (2.13)	22.90 (2.02)	21.42 (1.62)	21.50 (1.38)
Gender ratio (m:f)	9:3	7:5	11:1	7:5
Education (years)	15.75 (1.22)	15.79 (1.30)	15.04 (1.77)	14.50 (2.31)
BDI-11⁎	3.25 (3.92)	7.67 (7.10)	2.75 (1.81)	15.75 (12.95)
SPQ	9.25 (12.66)	22.83 (11.84)	10.58 (7.07)	22.80 (17.07)
STAI	35.67 (10.29)	41.67 (8.19)	33.00 (6.63)	42.58 (10.25)
Personal diagnosis of mental health problems	0	0	0	2 Depression, 1 ADHD
Familial diagnosis of mental health problems	1 Bipolar	0	1 Depression	1 Depression
Familial diagnosis of substance use problems	0	1 Alcohol	1 Crack, 1 Other	2 Alcohol
Spot the word task	51.17 (5.13)	49.75 (4.37)	51.42 (4.89)	48.75 (4.94)
Cannabis (*N*)	12	12	12	12
Cannabis used (years)	5.88 (3.48)	6.91 (3.00)	5.92 (2.15)	5.33 (2.39)
Cannabis use (days/month)	11.92 (6.84)	11.71 (10.24)	24.38 (9.06)	26.00 (5.64)
Days since last use	2.50 (1.38)	13.83 (33.64)	4.66 (8.15)	1.92 (0.79)
Time to smoke 3.5 g (days)	11.50 (15.83)	20.54 (16.13)	7.52 (8.84)	3.92 (2.75)
Alcohol (*N*)	12	12	12	12
Alcohol used (years)	6.04 (2.18)	6.71 (2.66)	6.50 (2.19)	5.25 ( 7.85)
Alcohol (days/month)	11.54 (5.66)	8.04 (4.87)	10.00 (7.67)	11.12 (7.43)
Tobacco (*N*)	6	9	10	9
Tobacco used (years)	4.57 (1.90)	5.22 (2.54)	5.50 (2.37)	5.83 (3.02)
Tobacco (days/month)	20.00 (11.40)	22.45 (12.16)	23.80 (10.89)	27.56 (7.33)
Tobacco cigarettes (day)	6.66 (3.77)	6.39 (3.12)	8.55 (5.31)	9.22 (4.47)

⁎ 1 Person׳s BDI is missing and 1 person has been replaced with the mean.
